# An In Silico In Vitro and In Vivo Study on the Influence of an Eggplant Fruit (*Solanum anguivi* Lam) Diet on Metabolic Dysfunction in the Sucrose-Induced Diabetic-like Fruit Fly (*Drosophila melanogaster*)

**DOI:** 10.3390/foods13040559

**Published:** 2024-02-12

**Authors:** Esther Nwanna, Roseline Ojo, Nusrat Shafiq, Awais Ali, Emmanuel Okello, Ganiyu Oboh

**Affiliations:** 1 Functional Foods and Nutraceutical Research Unit, Department of Biochemistry, Federal University of Technology, Akure 340252, Nigeria; 2 Veterinary Medicine Teaching and Research Center, University of California, Tulare, CA 93274, USA; 3 Synthetic & Natural Product Discovery Laboratory, Department of Chemistry, Government College Women University, Faisalabad 38000, Pakistan; 4 Department of Biochemistry, Abdul Wali Khan University Mardan, Mardan 23200, Pakistan

**Keywords:** *Solanum anguivi* lam, ripe fruit, metabolic dysfunctions, inflammation, enzymes, food security, bioactive compounds, hesperidin

## Abstract

**Introduction:** Africa faces immense food and health insecurity challenges, a problem partly attributed to food loss and waste during postharvest handling and distribution. In the context of research to meet the sustainable development goals, this project specifically addressed the postharvest loss of the ripe indigenous eggplant (*Solanum anguivi* lam) fruit called “Igba Yinrin” by Yoruba in South-West Nigeria, which is usually discarded in farms. The study was carried out on ripe and unripe fruits to better understand their value by comparing their effects in diabetes treatment. **Methods:** The study sought to assess the effects of a diet including ripe or unripe mature eggplant fruits in the sucrose-induced diabetic-like fruit fly. Bioactive compounds were identified and quantified with HPLC–UV, while the antioxidant vitamin (A, C, E), carotenoid, and mineral (Na, K, Ca, Mg, Fe, P, and Zn) content was analyzed in the fruits. Extracts were used to investigate their in vitro anti-inflammatory properties on cyclooxygenases (COX 1 and 2), 5-lipoxygenase (5-LOX), and anti-diabetes enzymes [α-amylase and α-glucosidase], while extract-supplemented diets (0.25–1% concentration) were fed to the fruit flies for 14 days. **Results:** Interestingly, the results showed that the ripe fruits had a significantly (*p* < 0.05) higher total phenol and flavonoid content, as well as a higher content of vitamins, carotenoids, and minerals, than the unripe fruits. The in vivo activities of antioxidant enzymes [superoxide dismutase (SOD), catalase (CAT), and glutathione transferase (GST)] and the total thiol level increased, while the blood glucose, reactive oxygen species (ROS), and malondialdehyde (MDA) levels decreased in *Drosophila melanogaster* (fruit fly). An in silico docking analysis showed strong binding affinity of the above-mentioned enzymes under investigation with the ligands hesperidin, naringin, and myricetin, which are bioactive compounds contained in the examined extracts. **Conclusions:** There was no significant difference in the biological effects of the ripe and unripe fruit extracts on inflammatory and anti-diabetes enzyme activities, which means that the ripe fruit, usually discarded, could serve as a sustainable alternative source of food nutrients.

## 1. Introduction

Diabetes is a multifaceted metabolic disorder affecting carbohydrate, fat, and protein metabolism. It is caused by increased levels of circulating blood sugar and insulin deficiency characterized by abnormal insulin secretion and insulin resistance in the body [1]. Type-2 diabetes (T2D) affects 80% of those living with diabetes and is largely due to an unhealthy diet and a sedentary lifestyle, which might lead to persistent high glucose levels in the blood, oxidative stress, aging, and other metabolic imbalances [1]. 

The use of the non-vertebrate organism *Drosophila melanogaster* as a model tool for research on various human diseases is important because this fly has biochemical features and characteristics similar to those of mammals; therefore, its use is increasing [2]. This model organism has been tested and has gained worldwide reputation for use in biomedical research, such as in diabetes studies and in other studies involving genetic manipulations [3,4]. Certain reports identified that disease-causing genes in humans are conserved in *Drosophila melanogaster*, such as those associated with the insulin-like growth factor signaling (IGFS) pathway. About 70–75% of the brain cells in the fruit fly are insulin-producing cells (IPCs) that are similar to the vertebrate pancreatic β cells and secrete seven insulin-like peptides (ILPs) [5,6,7]. In addition to this, using the fruit fly for research studies is easy and cost-effective, since it is not expensive to maintain them. 

The indigenous eggplant (*Solanum anguivi* lam) belongs to the Solanaceae family and is locally called “Igba Yinrin” by the Yoruba people in South-West Nigeria. It is commonly known as a forest bitter berry and a non-tuberous and highly polymorphic indigenous medicinal fruit that is widely distributed in non-arid areas of Africa and has nutritional and therapeutic potential [8,9]. A study reported that ripe and the unripe (green) eggplants are used in folklore medicine. These eggplants is also used in Ghana and Cameroon cuisine as one of the ingredients of a dish called “Nkwi”. Despite the benefits of the unripe fruits being known, no study has reported the properties of the ripe fruit (with an orange coloration) which is usually left to waste and rot in farms. The ripe eggplant fruits are usually discarded partly due to their low acceptability and the ignorance of their benefits and thus are among the post-harvest food crops lost after harvest in Nigeria, West Africa. Meanwhile, a study [10] showed that ripening brings changes in the fruit content of phenolic compounds, organic acids, and carbohydrates, as well as in its color, texture, and flavor [10]. 

Nigeria is facing an immense food insecurity challenge, a problem partly attributed to the increasing loss of food crops during post-harvest handling and distribution to retail markets. The reduction in post-harvest loss of this eggplant fruit and its use as a food ingredient for functional food development, such as in cookies and dumplings, could promote sustainable food security and a better health treatment/management of non-communicable diseases (NCDs) and cardiovascular diseases (CVDs), a task that requires an integrated approach (healthy diet and lifestyle). Therefore, this research was carried out on both ripe and unripe eggplants to compare their biological effects, with a focus on the anti-inflammatory, antioxidant, and anti-diabetic properties of a diet containing ripe *Solanum anguivi* lam fruits in diabetic *Drosophila melanogaster* (fruit fly).

## 2. Materials and Methods

### 2.1. Sample Collection

Ripe and unripe eggplant (*Solanum anguivi* lam) fruits were obtained from the botanical garden of the FUTA, Akure, as shown in Figure S1 (Supplementary File). The identification and authentication of the fruits were performed at the FUTA Herbarium; and were recorded with number 0291a and 0291b. Common fruit processing methods were adopted, including cleaning, sorting, cutting, pulping, drying, and grinding the fruits as needed [11]. The fruits were processed according to the method described by [12].

### 2.2. Mineral and Vitamin Determination

Mineral elements (Na, K, Ca, Mg, Fe, P, and Zn) were analyzed according to AOAC [13] and Perkin-Elmer [14]. The method described in [15] was used to determine the total carotenoid content, while vitamin C determination was carried using a method reported in [16], vitamin A content was determined according to [17], and vitamin E content was obtained according to [18]. HPLC–UV [high-performance liquid chromatography with ultraviolet detection] was carried out on the samples as described in [19]. This is an advanced technique used for separating and quantifying bioactive compounds in a mixture. In this method, a liquid sample is loaded in a column, and its components are detected by their absorption of ultraviolet light, which provides quantitative and qualitative information. The standard compounds in Table 1 were investigated and identified according to a standard protocol.

### 2.3. Preparation of Aqueous Extracts of Eggplant Fruits

The method used to prepare eggplant fruit extracts for the analyses was previously described [20]. The eggplant fruits were washed, cut, and then blended into a puree. The puree was filtered through cheesecloth to collect the liquid, which was the eggplant extract and was freeze-dried.

### 2.4. Total Phenol Content

The total phenol content in aqueous samples was determined using the Folin–Ciocalteu assay [21]. The samples were mixed with diluted Folin–Ciocalteu reagent and sodium carbonate and left react. The blue complex mixture was measured at 750 nm. With a calibration curve obtained using gallic acid, we determined the total phenolic content, expressed as milligram of gallic acid equivalents (GAEs).

### 2.5. Total Flavonoid Content

The method reported in [22] was used for the total flavonoid content determination. Using a suitable solvent to extract the sample, we obtained a flavonoid–AlCl_3_ complex [22]. The absorbance of the complex was measured at a certain wavelength, and the flavonoid concentration was calculated using a standard curve. Quercetin was used as a standard, and the flavonoid content was expressed in terms of quercetin equivalent (QUE).

### 2.6. In Vitro Enzyme Assays

#### 2.6.1. Assay for Cyclooxygenase Activity

The method described in [23] was used to assess the extract effect on cyclooxygenase (COX1 and COX2) activities. It typically involves measuring the enzyme activity in the context of the arachidonic acid cascade. This often includes using specific substrates and detecting the production of related metabolites. The assay can be conducted using various techniques, such as enzyme immunoassays, radioactive assays, or liquid chromatography coupled with mass spectrometry.

#### 2.6.2. Assay for 5-Lipoxygenase Activity

The assay to determine 5-lipoxygenase activity involves measuring the conversion of arachidonic acid to leukotriene. This is typically achieved by incubating the enzyme with the substrate, arachidonic acid, and assessing the production of leukotriene products, often using techniques like high-performance liquid chromatography (HPLC) or enzyme immunoassays. The method described by Viji and Helen [23] was used to assess the extracts’ effect on lipoxygenase. 

#### 2.6.3. α-Amylase Assay

The phenolic aqueous extract was used to determine the percentage (%) of α-amylase enzyme inhibitory activity [24]. The α-amylase assay is a method for quantifying the activity of the enzyme α-amylase, which breaks down starch into simpler sugars. This assay typically involves mixing the enzyme with a starch substrate, letting it react, and then measuring the conversion of starch to reducing sugars using colorimetric reagents, often with spectrophotometry.

#### 2.6.4. α-Glucosidase Assay

The α-glucosidase assay is a method used to measure the activity of the enzyme α-glucosidase, which catalyzes the hydrolysis of disaccharides into glucose. This assay involves incubating the enzyme with a substrate like p-nitrophenyl-α-D-glucopyranoside (pNPG) and then measuring the release of p-nitro phenol, typically using spectrophotometry, as an indicator of enzymatic activity. The phenolic aqueous extract was used to determine the percentage (%) of α-glucosidase enzyme inhibitory activity [25].

### 2.7. In Vivo Study

Wild-type *D. melanogaster* Harwich strain flies were reared using the method described by Oyeniran et al. in the *Drosophila* Laboratory unit of FUTA [5]. This method indicates the temperature, humidity, and nutrition conditions to preserve the wild-type strain. For best health and reproduction, we used suitable containers with standard Drosophila food medium, controlling the population density and providing fresh medium on a regular basis.

#### 2.7.1. Feed Formulation and Survival Study

Feed formulation, survival study, toxicological assays, and evaluation of locomotor performance using negative geotaxis were carried out using the method described in [5], with ripe and unripe dried eggplant flour included in the formulated food.

#### 2.7.2. Experimental Design and Biological Assays

The choice of the eggplant flour concentration in the formulated food (0.25% to 1%) was based on a preliminary study on fly survival rate where various concentrations between 0.1 and 2% were used. Flies 3–5 days old were separated into 8 groups, with 40 flies per vial. The experimental groups and design are shown in Table 2. The incubator was set at 25 ± 1 °C. The fruit flies were sacrificed, and the obtained tissue homogenates were extracted. The tissue extracts were used to carry out in vivo assays for the determination of protein content [26], α-amylase activity (Worthington, Biochemical Corp (1978) [24], α-glucosidase activity [25], glucose level—using the Sigma Randox kit and expressing it in mg/dL—ROS level [27], MDA content [28], total thiol [29], CAT activity [30], GST activity [31], and SOD activity [32].

### 2.8. In Silico Study

The method of Pettersen [33] was used to dock the 3-dimensional crystal structures of the protein targets with the PDB IDs Cox-1 6Y3C (1), Cox-2 5F1A (2), lipoxygenase 4NRE (3), α-amylase 1HNY (4), and α-glucosidase 5NN8 (5), in the protein database (PDB) (www.pdb.org/pdb, accessed on 1 March 2023) and the ligands, that is, the phenolic compounds identified in the HPLC–UV analysis (Table 1), using a computational docking study system, as described by Umar et al. [34].

### 2.9. Data Analysis

All statistical analyses were carried out using the software Graph pad PRISM (Version.6.0) in triplicate readings, with *p* < 0.05 indicating statistical significance [35]. 

## 3. Results

### 3.1. Phenolic Characterization Using HPLC–UV

As shown in Table 1 and Supplementary Figures S2 and S3, it was found that the ripe fruits contained considerable amounts of phenolic compounds such as phenol, cinnamic acid, quercetin, chlorogenic acid, and myricetin. Ripening increased the concentration of some compounds (rutin, gallic acid, catechin, syringic acid, isoquercitrin, piperic acid, hesperidin, and naringin). All the polyphenols found in the ripe fruits were also found in the unripe fruits. The ripe fruits had a total phenolic content of 2205.43 mg/100 g, while the unripe fruits contained 2354.94 mg/100 g of phenolic compounds. This suggests that the ripe fruits could be as useful as the unripe fruits and should not be discarded. This clearly shows how useful nutrients which could be utilized in food products’ development are wasted in farms due to lack of information.

### 3.2. Vitamins and Minerals 

The results of the analysis of essential minerals and vitamins, shown in Table 3 and Table 4, revealed significantly higher levels (*p* < 0.05) of some minerals and of vitamins A, C, and E in the ripe fruits than in the unripe fruits; we measured the following mineral levels: sodium, 54.1 mg/kg, potassium, 82 mg/kg, magnesium, 53.1 mg/kg, iron, 7.012 mg/kg, and zinc, 3.052 mg/kg in the ripe fruits. These results clearly show that ripening gradually increased the content of some of the mineral elements in the eggplant fruits.

### 3.3. Phenolic and Flavonoid Content

Table 5 shows the in vitro determined phenolic and flavonoid content in ripe and unripe fruits. The unripe fruits had a phenolic content of 8.64 ± 1.92 mg GAE/100 g and a flavonoids content of 1.69 ± 0.15 mg QUE/100 g, and the ripe fruits showed a significantly higher (*p* < 0.05) phenolic content (12.9 ± 1.05 mg GAE/100 g) and flavonoid content (2.46 ± 0.12 mg QUE/100 g). 

### 3.4. Cyclooxygenass (COX), Lipoxygenase (5-LOX), α-Amylase, and α-Glucosidase Activities

COX 1 and 2 and 5-LOX are key enzymes in the arachidonic acid (AA) metabolism, linked to inflammation. Unripe fruits showed lower (%) cyclooxygenases (45.93 ± 1.38) and lipoxygenase (35.82 ± 1.03) inhibition activity compared to ripe fruits, which displayed significantly higher (%) inhibition of cyclooxygenases (57.17 ± 0.69) and lipoxygenase (46.20 ± 1.24). There was no significance difference (*p* = 0.05) in the effect of the ripe and unripe fruit extracts on glucosidase as shown in Table 6.

### 3.5. Effect of the Ripe and Unripe Eggplant-Inclusive Diets on Weight and Movement

**Figure 1:**
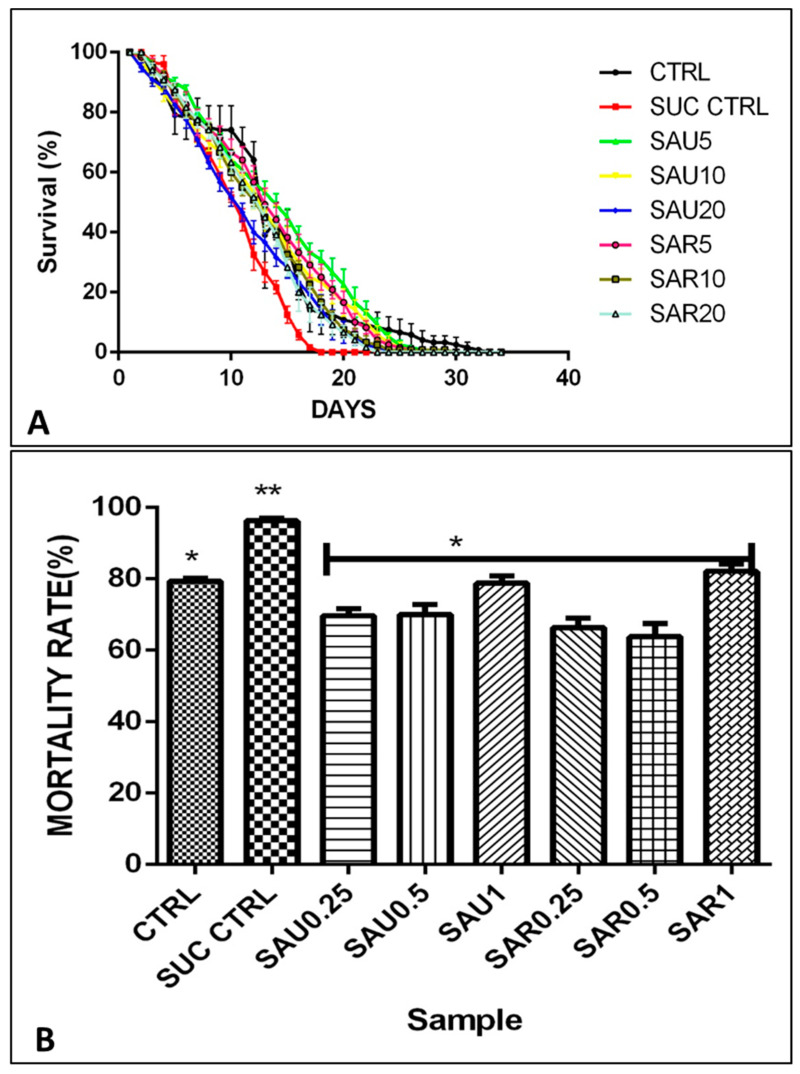
(**A**) % Survival rate of the fruit flies in days after treatment with different supplement concentrations. (**B**) % Mortality of the fruit flies. Different symbols (*, **) indicate significant differences (*p* < 0.05) between the treated groups (using various supplement concentrations), the sucrose-treated group, and the positive control group.

Eight groups (40 flies in one vial) of sucrose-induced diabetic fruit flies were fed a diet that included eggplant flour from ripe or unripe fruits at a concentration of 0.25–1%, as shown in the experimental design (Table 2). Figure 1A,B show the survival rate and mortality rate of the fruit flies treated with the inclusive diet. The survival rate was significantly higher for the treated fruit flies than for the untreated fruit flies in group 2. The flies in group 2 gained weight because of fat accumulation due to insulin resistance and lipid build-up. The unripe-fruit diet had a better effect on weight loss than the ripe-fruit diet. The movement of the flies was consistent with the survival and mortality results (Figure 1A); group 2 (untreated group) had a higher mortality rate compared to group 1 (Figure 1B). 

### 3.6. Effect of the Ripe and Unripe Eggplant-Inclusive Diets on α-Amylase and α-Glucosidase 

**Figure 2:**
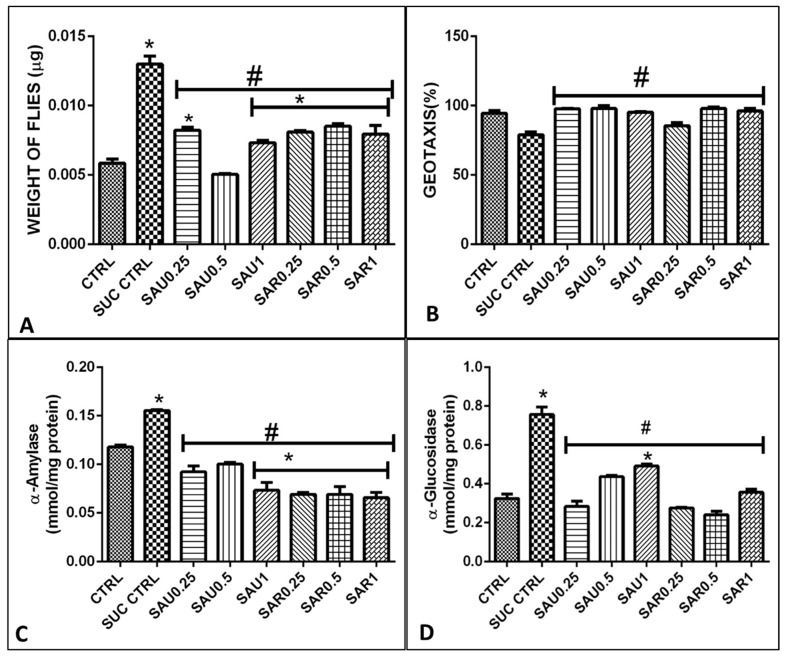
Different symbols (*, #) indicate significant differences (*p* < 0.05) between the treated groups (using various supplement concentrations), the sucrose-treated group, and the positive control group. (**A**). Effect of the eggplant-supplemented diet on the weight of the flies. (**B**) Effect of the eggplant-supplemented diet on negative geotaxis (**C**). Effect of the eggplant-supplemented diet on α-amylase activity (**D**). Effect of the eggplant-supplemented diet on α-glucosidase activity.

A reduction was observed in amylase and glucosidase activities in groups 3 and 4 compared to the diabetic group 2 (Figure 2C). The reduction in enzyme activity was observed when using the supplements at a concentration in the range of 0.25–1% (groups 3–8). Amylase and sucrase have been reported to be the two most important carbohydrate-hydrolyzing enzymes in *Drosophila melanogaster*, as observed also in this study; likewise, glucosidase activity was found to be important (Figure 2D).

### 3.7. Effect of the Ripe and Unripe Eggplant Diets on the Glucose Level

**Figure 3:**
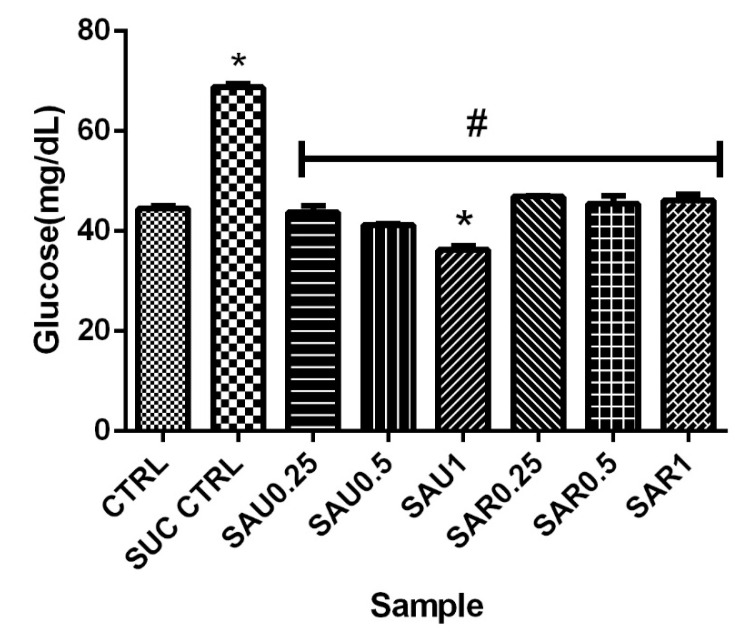
The effect of the ripe and unripe fruit diets on the glucose level. Different symbols (*, #) indicate significant differences (*p* < 0.05) between the treated groups (using various supplement concentrations), the sucrose-treated group, and the positive control group.

The results revealed an increase in the glucose level in the blood of the untreated diabetic flies compared to the diabetic flies receiving the eggplant-inclusive diet (groups 3–8); however, there was no significant difference in blood glucose level between the ripe eggplant diet- and the unripe eggplant diet-treated groups (Figure 3). 

### 3.8. Effect of the Eggplant Diets on ROS, MDA, and Total Thiols

**Figure 4:**
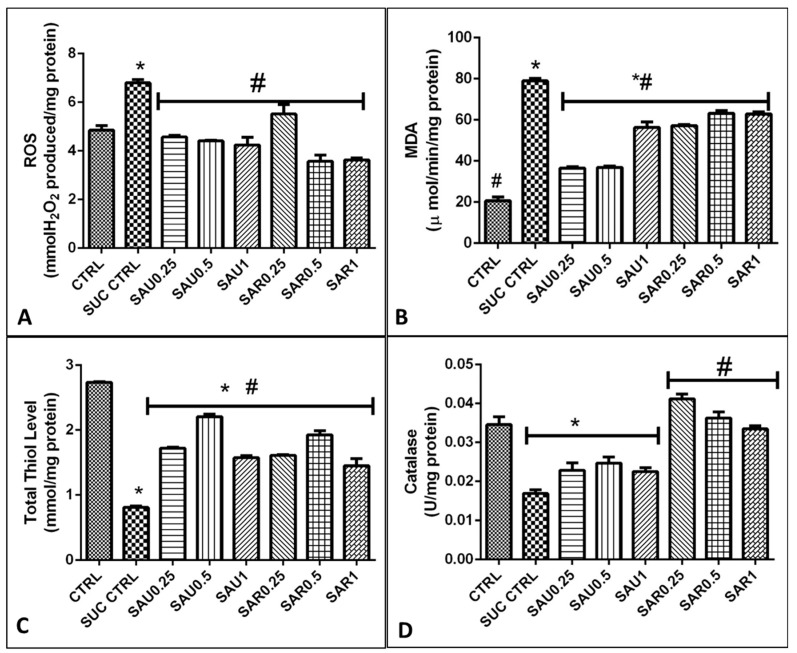
Different symbols (# and *) indicate significant differences (*p* < 0.05) between the treated groups, and the negative and positive control groups. (**A**) Effect of the eggplant-supplemented diet on the ROS level. (**B**) Effect of the eggplant-supplemented diet on MDA production. (**C**) Effect of the eggplant-supplemented diet on the total thiol level. (**D**) Effect of the eggplant-supplemented diet on CAT activity.

An increase in reactive oxygen species (ROS) such as (OH−, NO−, O−) radicals is related to the production of malondialdehyde (MDA) via lipid peroxidation. In fact, high ROS levels were associated with a high level of MDA (Figure 4A–C). There was a significant reduction of (*p* < 0.05) in the MDA level in the treated groups (3–8), which were administered the inclusive diet, relative to the groups 1 and 2. In particular, of great importance is the significant reduction obtained with the diet containing unripe *Solanum anguivi* lam at a concentration of 0.5%, which appeared to be more efficient than the diets containing the other concentrations.

### 3.9. Effect of the Eggplant Diets on Endogenous Antioxidants in a Cell-Based System

**Figure 5:**
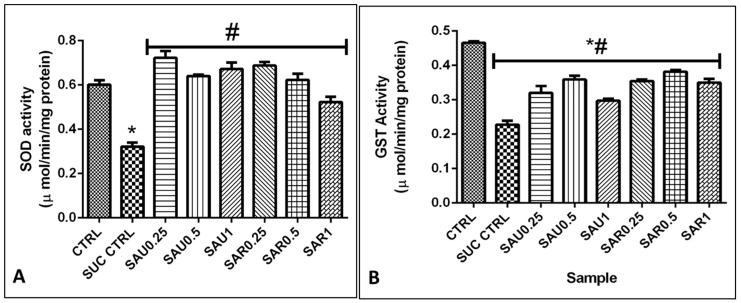
(**A**) Effect of ripe and unripe eggplant fruit diets on SOD activity. (**B**) Effect of the eggplant-supplemented diet on GST activity. Different symbols (# and *) indicate significant differences (*p* < 0.05) between the treated groups.

The results showed the same trend for the effect of the eggplant-supplemented diet on the activity of the antioxidant enzymes GST, SOD, and CAT, expressed in U/mg protein, in the flies (Figure 4D and Figure 5B). There was no difference in the activity of GST and SOD between the ripe and the unripe fruit diets, but CAT activity was higher in all groups given the ripe eggplant-inclusive diet (Figure 5). 

### 3.10. In Silico Results

**Figure 6:**
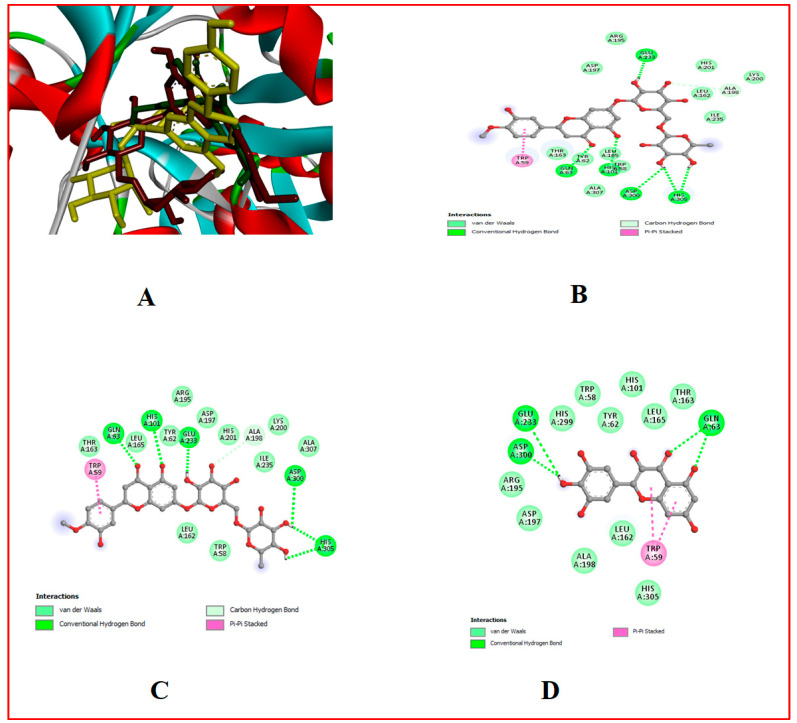
Molecular docking evaluation of hit compounds against α-amylase (1HNY). (**A**) The binding conformation of hesperidin (brown), naringin (yellow), and myricetin (green) in the binding domain of α-amylase (1HNY) shown in a 3D model. Molecular interaction analysis between the amino acid residues (side chains) within the binding domain and the atoms of (**B**) hesperidin, (**C**) naringin, and (**D**) myricetin in a 2D model. Docking was achieved through Auto Dock Vina, while 2D and 3D rendering was possible through the aid of BIOVIA’s Discovery Studio 2016.

**Figure 7:**
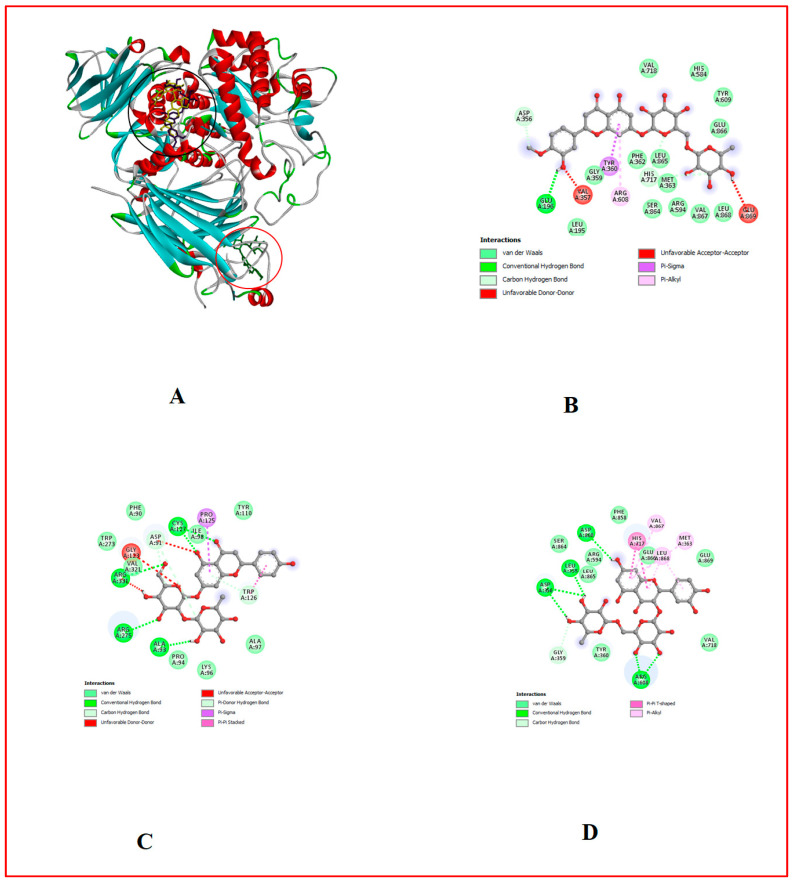
Molecular docking evaluation of hit compounds against α-glucosidase (5NN8). (**A**) The binding conformation of hesperidin (purple), naringin (yellow), and rutin (green) in the binding domain of α-glucosidase (5NN8) is shown in a 3D model. Molecular interaction analysis between the amino acid residues (side chains) within the binding domain and the atoms of (**B**) hesperidin, (**C**) naringin, and (**D**) rutin shown in a 2D model. Docking was achieved through Auto Dock Vina, while 2D and 3D rendering was possible through the aid of BIOVIA’s Discovery Studio 2016.

**Figure 8:**
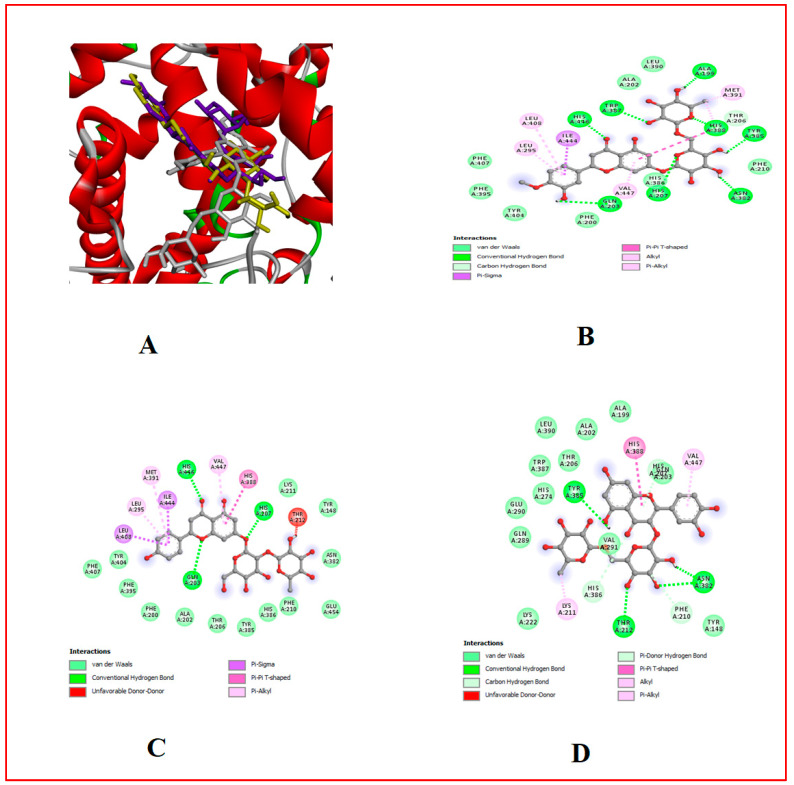
Molecular docking evaluation of hit compounds against Cox-1 (6Y3C). (**A**) The binding conformation of hesperidin (purple), naringin (white), and rutin (yellow) in the binding domain of Cox-1 (6Y3C) is shown in a 3D model. Molecular interaction analysis between the amino acid residues (side chains) within the binding domain and the atoms of (**B**) hesperidin, (**C**) naringin, and (**D**) rutin, shown in a 2D model. Docking was achieved through Auto Dock Vina, while 2D and 3D rendering was possible through the aid of BIOVIA’s Discovery Studio 2016.

**Figure 9:**
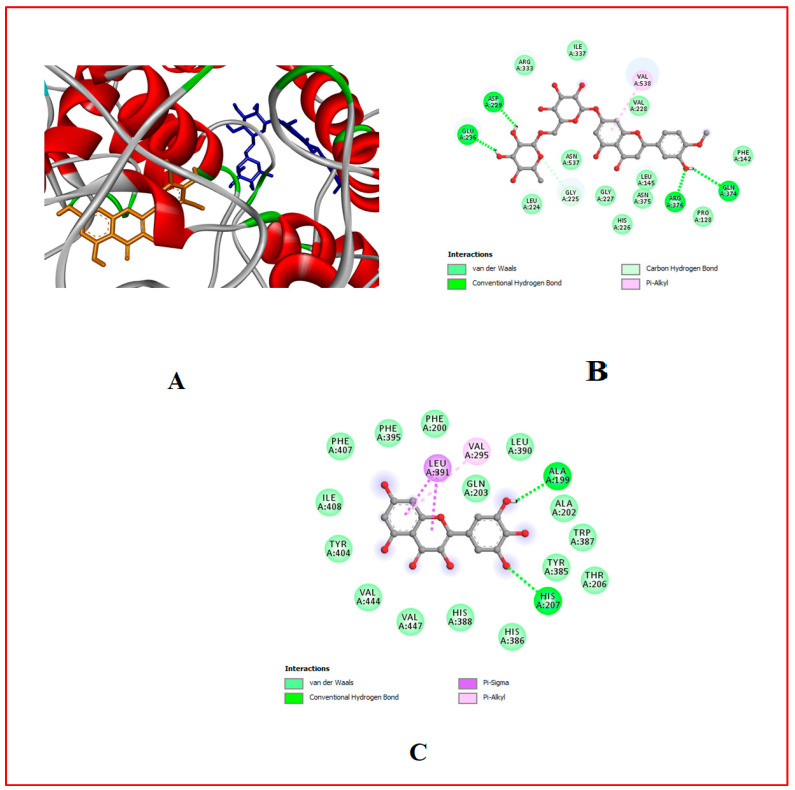
Molecular docking evaluation of hit compounds against Cox-2 (5F1A). (**A**) The binding conformation of hesperidin (blue) and myricetin (orange) in the binding domain of Cox-1 (6Y3C) is shown in a 3D model. Molecular interaction analysis between the amino acid residues (side chains) within the binding domain and the atoms of (**B**) hesperidin and (**C**) myricetin, shown in a 2D model. Docking was achieved through Auto Dock Vina while 2D and 3D rendering was possible through the aid of BIOVIA’s Discovery Studio 2016.

**Figure 10:**
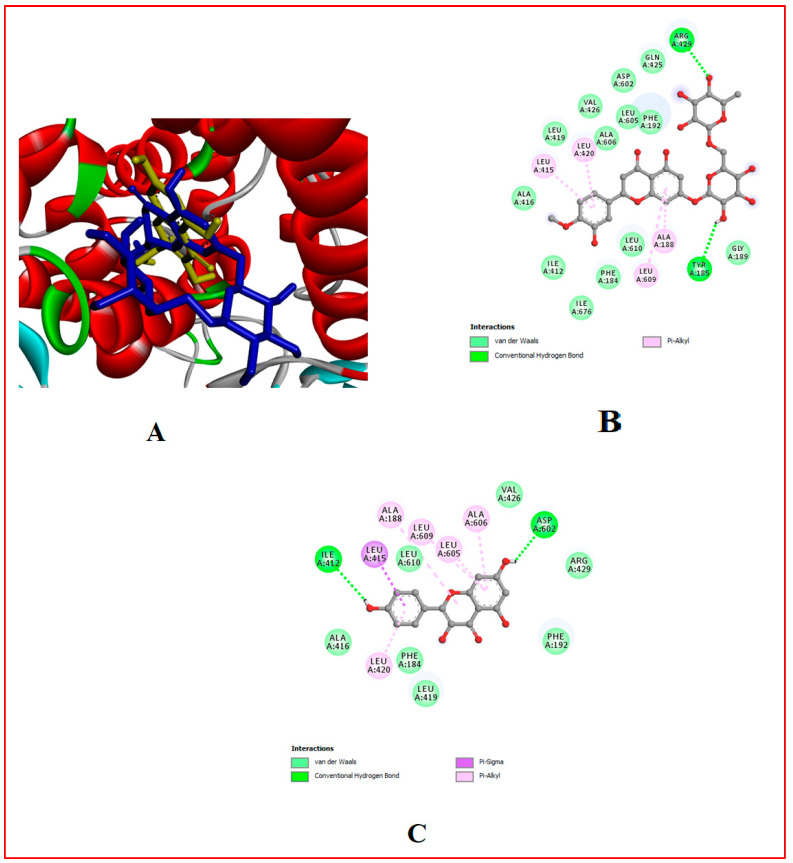
Molecular docking evaluation of hit compounds against lipoxygenase (4NRE). (**A**) The binding conformation of hesperidin (blue) and kaempferol (yellow) in the binding domain of lipoxygenase (4NRE) is shown in a 3D model. Molecular interaction analysis between the amino acid residues (side chains) within the binding domain and the atoms of (**B**) hesperidin and (**C**) kaempferol, shown in a 2D model. Docking was achieved through Auto Dock Vina, while 2D and 3D rendering was possible through the aid of BIOVIA’s Discovery Studio 2016.

The binding affinity of the bioactive compounds that are the ligands of Cox-1, Cox-2, 5-LOX, α-amylase, and α-glucosidase was investigated. It was found that relative to the control anti-diabetes drug (Acarbose) and anti-inflammatory (Ibuprofen) drugs used, out of the 17 ligands used for docking, hesperidin showed the highest binding affinity for all target proteins, followed by naringin and myricetin (Table 1, Table 7 and Table 8, Figure 2, Figure 3, Figure 4, Figure 5, Figure 6, Figure 7, Figure 8, Figure 9 and Figure 10). 

## 4. Discussion

This study compared the effects of ripe and unripe fruit diets on diabetes in order to evaluate their biological potential; however, our main focus was on the effects of ripe fruits, which are not always reported in studies because ripe fruits are considered waste. Due to ripening, about 60% of indigenous eggplant *Solanum anguivi* lam (SA) fruits in Nigeria are left to waste in farms, because the ripe fruit is given little or no importance. Meanwhile, this unwanted fruit is rich in viable phytochemicals, as reviewed in [36], which explained the benefits of phyto-compounds such as hesperidin—known as a bioflavonoid with anti-inflammatory, skin-lightening, antimicrobial, and wound healing properties—and naringin—which was found to have broad pharmacological potential [37]. In addition, myricetin was previously reported to have neuroprotective, analgesic, antihypertensive, antidiabetic. and hypolipemic potential [38]. 

This clearly shows that many beneficial bioactive compounds from nature are wasted yearly due to the early ripening of fruits and vegetables as well as inadequate/poor storage facilities. This present study showed that the ripe fruit contains more vitamins and minerals than the unripe one, which are essential for the proper functioning of the body. Vitamins are essential nutrients that are needed in the body and are involved in cell regulation, body metabolism, growth, and development, physiological processes, such as iron absorption, and the immune response [39]. We found that the levels of vitamins A, C, E, and carotenoids and of some minerals increased with ripening, which confirmed a previous report [11]; the change in coloration of the *Solanum anguivi* lam fruit could be due to the synthesis of alpha-carotene and beta-carotene, precursors of vitamin A [40].

COX and 5-LOX metabolic products such as prostanoids and leukotrienes are implicated in degenerative diseases such as cancer progression and may represent important targets for cancer chemoprevention or treatment [41]. From the in vitro results, the bioactive compounds present in the ripe eggplant extract could serve as dual inhibitors of inflammatory enzymes, able to block the COX and the 5-LOX metabolic pathways better than synthetic drugs (sulindac and aspirin). 

The ripe eggplant extrac bioactive compounds, as shown from the results, have either additive or synergistic effects on the two major enzymes in arachidonic acid metabolic pathways, thus inhibiting their inflammatory activities. Secondly, it could be suggested that eggplant extracts and related diet also possess another advantage, as they have small or no gastric toxicity, being natural products with a balancing effect compared with synthesized non-steroidal anti-inflammatory drugs such as sulindac and aspirin, which are commonly used but cause major side effects, such as ulcerative colitis, due to non-selective COX inhibition. This study clearly shows that the eggplant, especially if ripe, could display an enhanced anti-inflammatory potency based on polyphenol structure, which contains almost the same structural and functional groups as the above-mentioned drugs. This observation confirms the need to utilize natural products such as eggplant for non-selective dual COX/5-LOX inhibition without side effects [41,42]. 

Some reports indicated that some plant products such as curcumin, tea, silymarin, resveratrol, and green and black tea, rich in polyphenols, are used as natural dual enzyme inhibitors, which means that eggplant extracts or and its related diets could exert beneficial, therapeutic effects through the modulation of COX and LOX metabolic pathways [41,42]. In addition, it was also confirmed that chronic inflammation is implicated in the pathophysiology of diabetes, especially type-2 diabetes, and to that end, targeting inflammation may help ameliorate diabetes, preventing its sequence and other metabolic complications, which means that an eggplant-supplemented diet could exert multiple beneficial functions. 

Bioactive compounds such as vitamins, carotenoids, phenolic compounds, and flavonoids, which seem to be abundant in fruits, whether ripe or unripe, could be the reason behind the low weight gain, the increased survival, and the decreased mortality rate of the flies. This study confirmed what was reported in [5], i.e., that diabetic fruit flies administered a high-sucrose diet show obesity-linked lipogenesis caused by the repartition of fatty acids toward esterification, away from fatty acid β-oxidation, possibly by increasing malonyl-CoA. Moreover, there is a relationship between movement and weight gain which is also linked to obesity, meaning that a healthy lifestyle involving exercise and nutritious food rich in phyto-compounds, minerals, vitamins, and fiber, and with a low GI could holistically enable body wellness, as suggested by Nwanna et al. [12]. 

It was also observed that the ripe eggplant-inclusive diet was able to elicit positive effects better than the unripe fruit-inclusive diet, at the same time reducing the glucose, MDA, and thiol levels in the diabetic subjects compare to the untreated subject. There is connection between diabetes mellitus, oxidative stress, and reduced antioxidant activity [43], while the biomarkers of oxidative stress are related to reactive oxygen species, MDA, and thiols. Thiol is an organic compound containing a sulfur atom. It is also referred to as mercaptan [44]. Thiol acts as an antioxidant, stabilizing free radicals by accepting their unpaired electron. This means that a ripe eggplant diet, with its bioactive compounds, contains relatively more antioxidant molecules than an unripe eggplant diet [45].

Furthermore, an increase in endogenous antioxidant enzyme activities (GSH, CAT, and SOD) was observed in the groups treated with the supplemented diet, and diabetes causes tissue injury in flies as a result of the increased production of ROS [3]. The elevation in the activity of these enzymes might be a result of the scavenging ability of the bioactive compounds in the fruit diet, which eliminated the ROS species and exerted their effects by restoring the activity of antioxidative enzymes. SOD is an enzymatic antioxidant that defends against free radicals by converting superoxide anions to compounds with less damaging effects like hydrogen peroxide; it serves as the first-line defense mechanism against pro-oxidative injury in tissues, which is important for lifespan extension and survival of *Drosophila melanogaster* [3]. 

CAT is an enzyme that converts lesser damaging compounds such as peroxides and H_2_O_2_ to water and molecular oxygen [46]. CAT activity was reduced in the sucrose-treated and control groups; this could also be explained by the accumulation of H_2_O_2_. We found that the inclusive diet increased the activities of SOD and CAT, which suggests a quick response to fight free radicals by reducing the endogenous H_2_O_2_ that was generated and diminishing the toxic effects it could cause [46,47]. GST is needed to prevent damage and serves as a defense mechanism. The ability of the *Solanum anguivi* lam fruits, whether ripe or unripe, to restore the damaged antioxidant enzymes status in sucrose-induced diabetic flies cannot be overemphasized. 

It is interesting that the fruit contains high levels of bioflavonoid compounds such as hesperdin, naringin, and myricetin, with high binding affinity for all the proteins of interest in this study; these compounds could serve as good inhibitors of the enzymes associated with diabetes and inflammation, although it was earlier reported that they have numerous biological properties such as anti-microbial, anti-lipidemic, anti-cancer, and antioxidant properties [37]. Ripening increases the levels of some of these bioactive compounds, which further confirms the report of Nwanna et al. [9] of the immerse benefit of the eggplant fruit especially, the wild species. 

## 5. Conclusions

*Solanum anguivi* lam fruit ameliorated hyperglycemia and exerted anti-inflammatory effects in diabetic *Drosophila melanogaster* due to its richness in antioxidant vitamins, minerals, and bioactive compounds such as hesperdin, naringin, and myricetin. Hence, the above findings provide enough scientific evidence confirming that the ripe *Solanum anguivi* lam fruits, which are usually discarded, could serve as a viable source of nutrients. This study also shows that valuable vitamins, minerals, and phyto-nutrients are discarded in farms due to ignorance and could be alternatively converted to beneficial products for common use.

## Figures and Tables

**Table 1:** Characterization of polyphenol compounds in ripe (SAR) and unripe (SAU) *Solanum anguivi* fruits using HPLC–UV (mg/100 g).

Compounds	SAU (Unripe)	SAR (Ripe Fruit)
Rutin	2.36 × 10^−4^	5.47 × 10^−4^
Phenol	669.27	620.95
Vanillic acid	1.14 × 10^−5^	2.82 × 10^−5^
p-hydroxybenzoic acid	4.52 × 10^−6^	9.49 × 10^−6^
Cinnamic acid	117.55	110.46
Catechin	1.20 × 10^−2^	1.35 × 10^−2^
Gallic acid	7.96 × 10^−3^	9.21 × 10^−3^
Caffeic acid	1.19 × 10^−2^	1.26 × 10^−2^
Kaempferol	472.27	458.37
Syringic acid	6.33 × 10^−6^	7.08 × 10^−6^
Naringin	1.09 × 10^−6^	1.38 × 10^−6^
Piperic acid	3.17 × 10^−7^	3.88 × 10^−7^
Epicatechin	4.16 × 10^−4^	4.94 × 10^−4^
Quercetin	448.35	377.16
Myricetin	235.37	218.59
Chlorogenic acid	375.06	366.30
Isoquercitrin	2.94 × 10^−4^	3.19 × 10^−3^
Hesperidin	1.01 × 10^−3^	3.53 × 10^−3^
Total	2354.94	2205.43

**Table 2:** Experimental design of the eggplant-inclusive diet using ripe (SAR) and unripe (SAU) *Solanum anguivi* fruits.

Groups	Diets
Group 1	Basal only
Group 2	Sucrose-induced
Group 3	Sucrose-induced + Unripe sample (0.25%)
Group 4	Sucrose-induced + Unripe sample (0.50%)
Group 5	Sucrose-induced + Unripe sample (1%)
Group 6	Sucrose-induced + Ripe sample (0.25%)
Group 7	Sucrose-induced + Ripe sample (0.50%)
Group 8	Sucrose-induced + Ripe sample (1%)

**Table 3:** Mineral content in ripe (SAR) and unripe (SAU) *Solanum anguivi* fruits (mg/kg).

Sample	Na	K	Ca	Mg	Fe	P	Zn
SAU	46.80 ± 0.01 ^a^	78.01 ± 0.01 ^a^	29.02 ± 0.01 ^a^	47.50 ± 0.01 ^a^	4.71 ± 0.01 ^a^	8.31 ± 0.01 ^a^	2.95 ± 0.01 ^a^
SAR	54.10 ± 0.01 ^b^	82.01 ± 0.01 ^b^	23.50 ± 0.01 ^b^	53.10 ± 0.01 ^b^	7.01 ± 0.01 ^b^	7.83 ± 0.01 ^a^	3.05 ± 0.01 ^b^

The same letter in a column means no significant difference (*p* < 0.05). Values represent the mean ± standard deviation of replicate readings.

**Table 4:** Vitamin content in ripe (SAR) and unripe (SAU) *Solanum anguivi* fruits.

Sample	VIT A (mg/g)	VIT C (mg/g)	VIT E (mg/g)	Carotenoids (mg/g)
SAU	12.39 ± 4.81 ^a^	57.9 ± 0.2 ^a^	0.45 ± 0.07 ^a^	7.52 ± 0.101 ^a^
SAR	16.76 ± 4.73 ^b^	81.0 ± 1.4 ^b^	2.24 ± 0.06 ^b^	9.06 ± 0.022 ^b^

The same letter in a column means no significant difference (*p* < 0.05). Values represent the mean ± standard deviation of replicate readings.

**Table 5:** The total phenol content and total flavonoid content in ripe (SAR) and unripe (SAU) *Solanum anguivi* fruits.

Parameters	Total Phenol (mg GAE/100 g)	Total Flavonoids (mg QUE/100 g)
SAU	8.64 ± 1.92 ^a^	1.69 ± 0.15 ^a^
SAR	12.9 ± 1.05 ^b^	2.46 ± 0.12 ^b^

The same letter in a column means no significant difference (*p* < 0.05). Values represent the mean ± standard deviation of replicate readings.

**Table 6:** In vitro effects of ripe (SAR) and unripe (SAU) *Solanum anguivi* fruit extracts on cyclooxygenase and lipoxygenase activities (%) and IC_50_ of α-amylase and α-glucosidase.

Sample	Cyclooxygenases	Lipoxygenases	Alpha-Amylase	Alpa-Glucosidase
SAU	45.93 ± 1.38 ^a^	35.82 ± 1.03 ^a^	0.39 ^a^	0.82 ^a^
SAR	57.17 ± 0.69 ^b^	46.20 ± 1.24 ^b^	0.57 ^b^	0.81 ^a^

The same letter in a column means no significant difference (*p* < 0.05). Values represent the mean ± standard deviation of replicate readings.

**Table 7:** Grid box parameters selected during docking and binding sites of the selected protein targets.

S/N	Target Proteins	Center Grid Box (XYZ), Å	Dimension (XYZ), Å	Amino Acid Residues in the Binding Site
1.	Cox-1 (PDB code: 6Y3C)	−33.888 × −57.283 × −6.6438	55.567 × 35.2575 × 50.6242	Trp387, Val349, Ile523, Leu408, Leu295, Met391, Ile444, His446, Asp450, Glu454, Gly214, Tyr130, Cys41, Thr322, Pro218 and Ser126 (1)
2.	Cox-2 (PDB code: 5F1A)	43.047 × 36.6684 × 236.2794	25.928 × 48.9905 × 36.5275	Tyr385, Ser53, Leu531, Arg513, Tyr348, Val523, Trp387, Leu384, Val349, Ile124, Val344, His90, Arg120, Val434, Tyr355, Ser353, Glu524, Leu352, Phe518, Ala527, Phe201 and Lys248 (2)
3.	Lipooxygenase (PDB code: 4NRE)	12.2559 × −56.678 × −217.28	25.9561 × 23.3251 × 24.1261	Arg429, Leu605, Leu609, Leu419, Phe184, Leu415, Glu369, Phe438, Phe365, Thr431, Val603, Val426, Ala606, Asp602, Asn413, Leu607, Gln560, Val427, Leu610, Ile412, Ala416, His373, Leu374, Ile676 and Leu420 (3)
4.	α-Amylase (PDB code: 1HNY)	8.8333 × 46.3378 × 19.0379	25.7746 × 23.3197 × 22.1424	Glu233, Asp197, Asp300, His101, Gln63, His201, Tyr151, Arg195, Lys200, Val234, Ile235, His299, His305 and Ala307 (4)
5.	α-Glucosidase (PDB code: 5NN8)	−7.6385 × −34.298 × 85.0213	30.5278 × 64.2095 × 50.5598	Asp518, Asp616, Asp404, Asp518, Arg600, His674, Trp376, Ile441, Trp516, Met519, Trp613, Phe649, Asp282, Arg437, Gly435, His432, Gly434, Ala93, Asp91, Cys127, Pro125 and Trp126 (5)

**Table 8:** Molecular docking score of the examined compounds in terms of binding energy (kcal/mol).

Ligand	Binding Energy (kcal/mol)				
	Amylase	Glucosidase	Lipooxygenase	Cox-1	Cox-2
p-hydroxybenzoic acid	−5.3	−5.4	−5.6	−6.1	−6.0
Gallic acid	−5.8	−6.0	−5.8	−6.8	−6.5
Catechin	−8.6	−6.9	−8.1	−9.0	−7.8
Vanillic acid	−5.4	−5.6	−5.5	−6.4	−6.5
Hesperidin	−9.1	−9.5	−8.5	−9.6	−8.3
Syringic acid	−5.3	−5.9	−5.3	−6.1	−6.1
Epicatechin	−8.6	−6.9	−8.1	−9.0	−7.8
Naringin	−9.1	−8.4	−8.0	−9.8	−8.0
Cinnamic acid	−5.8	−5.8	−7.0	−6.4	−6.3
Caffeic acid	−6.4	−6.1	−6.2	−7.1	−6.6
Chlorogenic acid	−7.5	−7.0	−7.4	−8.7	−8.1
Quercetin	−8.6	−7.2	−8.1	−9.1	−8.1
Isoquercitrin	−7.8	−8.2	−7.5	−8.7	−7.5
Rutin	−8.2	−8.5	−7.3	−9.3	−6.7
Kaempferol	−8.5	−7.1	−8.5	−9.1	−7.7
Myricetin	−8.7	−7.3	−7.8	−9.1	−8.3
Piperic acid	−7.1	−6.4	−7.2	−7.3	−6.8
Acarbose *	−6.6	−7.8	−	−	−
Ibuprofen *	−	−	−7.2	−7.5	−7.3

* Control compounds for the target proteins.

## Data Availability

The original contributions presented in the study are included in the article, further inquiries can be directed to the corresponding author.

## Supplementary Materials

The following supporting information can be downloaded at https://www.mdpi.com/article/10.3390/foods13040559/s1, Figure S1: Ripe and Unripe Solanum anguivi lam (eggplant) fruits. Source FUTA Botanical Garden; Figure S2: Chromatogram of ripe *Solanum anguivi* lam fruit; Figure S3: Chromatogram of unripe *Solanum anguivi* lam fruit.
