# Supplementary material for: An In Silico In Vitro and In Vivo Study on the Influence of an Eggplant Fruit (Solanum anguivi Lam) Diet on Metabolic Dysfunction in the Sucrose-Induced Diabetic-like Fruit Fly (Drosophila melanogaster)
Source: Foods. 2024 Feb 12;13(4):559. doi: 10.3390/foods13040559 (PMC10888091; doi:10.3390/foods13040559)
Supplement: Supplementary file 1 [file foods-13-00559-s001.zip › foods-2743113-supplementary.pdf]

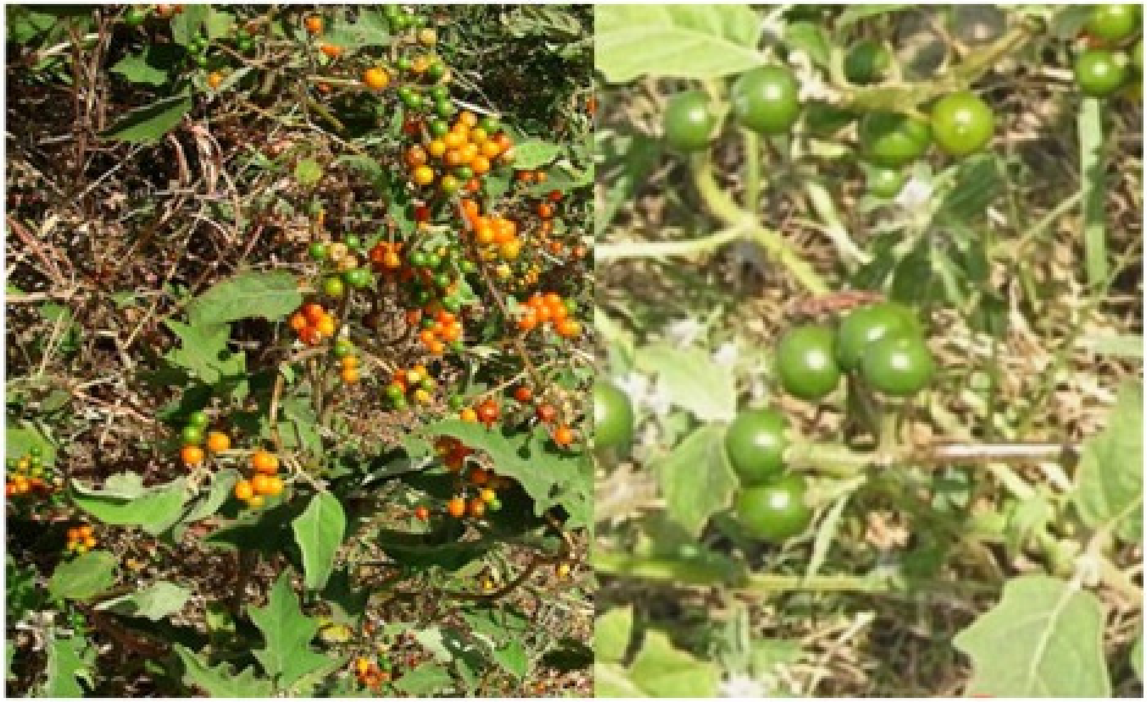

Ripe Unripe

Figure S1: Ripe and Unripe *Solanum anguivi* lam (eggplant) fruits. Source FUTA Botanical Garden.

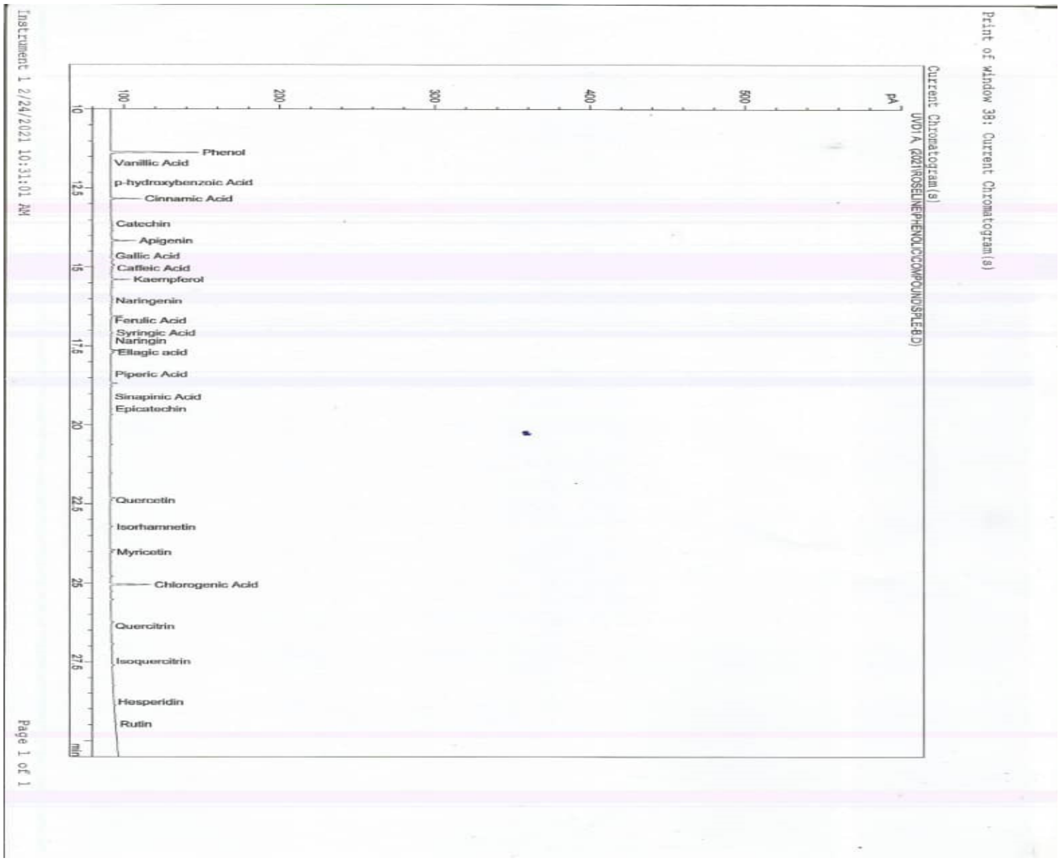

Figure S2: Chromatogram of ripe *Solanum anguivi* lam fruit.

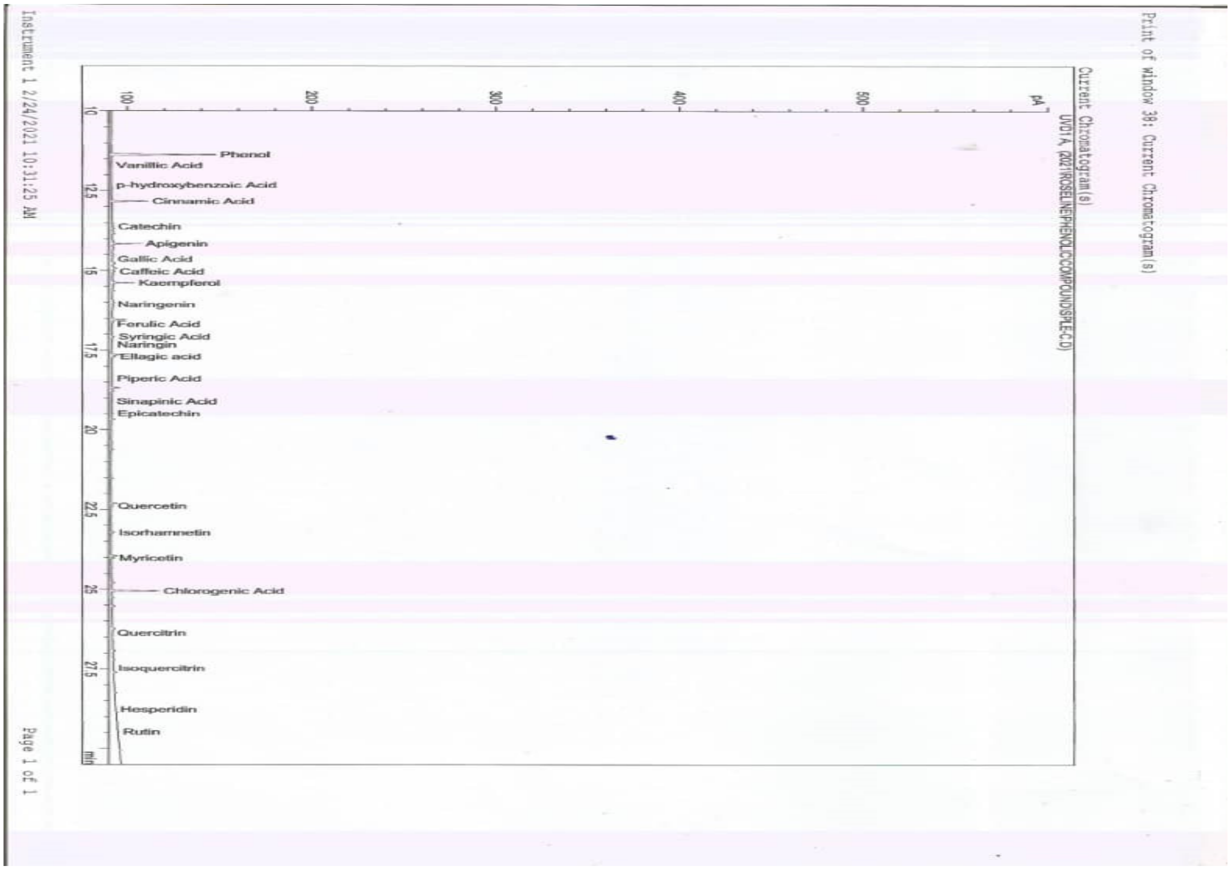

Figure S3: Chromatogram of unripe *Solanum anguivi* lam fruit.
